# Reduction in Perioperative Risk in Patients with Spinal Muscular Atrophy Following the Release of Disease-Modifying Therapies: An Analysis of the National Surgical Quality Improvement Program Database

**DOI:** 10.3390/children12091255

**Published:** 2025-09-18

**Authors:** Erin Toaz, Nisha Pinto, Keith Kilner, Eric Cheon

**Affiliations:** Department of Pediatric Anesthesiology, Ann & Robert H. Lurie Children’s Hospital of Chicago, Northwestern University, Chicago, IL 60611, USA; etoaz@luriechildrens.org (E.T.); npinto@luriechildrens.org (N.P.); kkilner@luriechildrens.org (K.K.)

**Keywords:** spinal muscular atrophy, SMA, nusinersen, spinraza, onasemnogene abeparvovec, zolgensma, postoperative pulmonary complications, length of stay

## Abstract

**Highlights:**

**What are the main findings?**

- A year following widespread implementation of new disease-modifying therapies, patients with SMA demonstrated a reduced risk for postoperative pulmonary complications and a reduction in length of stay at a population level compared to patients with SMA before the new treatment era.

**What is the implication of the main finding?**

- Improved motor outcomes and evolving clinical phenotypes with early treatment initiation may account for this trend.
- Multivariable prediction models must consider the dynamic effects of new therapies on the perioperative outcomes of affected populations.

**Abstract:**

**Background/Objectives:** Spinal muscular atrophy (SMA) is a progressive neurodegenerative disease resulting in proximal muscle weakness and paralysis. SMA treatment has radically changed in the past 10 years thanks to the development of novel therapies such as nusinersen and onasemnogene abeparvovec. Since the advent of new treatments, the incidence and perioperative risk factors of patients with SMA undergoing longer, higher-risk surgeries are unknown. We hypothesized that patients with SMA would be at an overall elevated risk for postoperative pulmonary complications (PPC) and prolonged length of stay compared to the general population, but that this would be reduced in patients undergoing surgery in the years after the release of new therapies. **Methods**: Patients who underwent surgery at a continuously enrolled American College of Surgeons National Surgery Quality Improvement Program-Pediatric hospital from 1 January 2012, to 31 December 2021, were included in this study. Cases with missing covariate or primary outcome data were excluded from the analysis. Patients with ages greater than 17 years, preoperative tracheostomy, preoperative mechanical ventilation, missing covariate or primary outcome data were excluded. Patients with SMA were identified by their ICD-9 and 10 codes. A cutoff year of 2018 was chosen for analysis of the primary outcomes as this was a full year after nusinersen received FDA approval. **Results**: On univariable analysis, the risk for PPC in patients with SMA was reduced in patients undergoing surgery in 2018 or later compared to pre-2018 (pre-2018 OR 4.44, 95% CI 1.56–9.6, *p* = 0.008; post-2018 OR 3.48, 95% CI 0.84–9.12, *p* = 0.08). On multivariable analysis, the association between SMA and PPC substantially decreased in 2018 and after but was no longer statistically significant (pre-2018 OR 1.96, 95% CI 0.80–4.80, *p* = 0.14; post-2018 OR 1.03, 95% CI 0.33–3.26, *p* = 0.96). SMA was positively associated with LOS in the pre-2018 cohort, with a coefficient from a log linear model of 0.67 (95% CI 0.32–1.01; *p* < 0.001), and SMA adding an additional 1.93 days in LOS. For data post-2018, the effect of SMA on LOS was no longer statistically significant. **Conclusions**: Utilizing a large dataset, we found a reduced association between SMA and PPC a year following widespread implementation of SMN antisense oligonucleotide therapy, and a statistically significant reduction in LOS in patients with SMA after 2018. This may reflect improved motor outcomes and respiratory mechanics in the new treatment era.

## 1. Introduction

Spinal muscular atrophy (SMA) is an autosomal recessive neuromuscular disease characterized by degeneration of alpha motor neurons in the spinal cord, resulting in progressive proximal muscle weakness and paralysis [1]. It is caused by mutations in the Survival Motor Neuron 1 (*SMN1*) gene on chromosome 5q13, leading to insufficient production of functional SMN protein. SMA is clinically classified into four phenotypes based on age of onset and motor milestones achieved, with the most severe phenotype, SMA type 1, accounting for more than half of all cases [1]. Untreated SMA is a leading genetic cause of death in young children [1,2].

Children with SMA frequently require anesthetic care for diagnostic testing and interventions such as enteral feeding tubes and orthopedic procedures. Given their baseline compromised respiratory mechanics, the anesthetic challenge is to ensure effective pain management and sedation while minimizing cardiorespiratory risks [3,4].

SMA treatment has radically changed over the past decade with the development of nusinersen (Spinraza), a novel antisense oligonucleotide drug administered as serial intrathecal injections and onasemnogene abeparvovec-xioi (Zolgensma), a gene replacement therapy, resulting in improved motor outcomes and expected lifespan of these patients [5,6,7]. Nusinersen received US Food and Drug Administration approval for treatment of SMA in December 2016 and onasemnogene abeparvovec in 2019 [2,8]. With the new treatments, the observed disease trajectories differ significantly from the known natural history of the disease, and the most impressive results have been observed when treatment is initiated before the first clinical symptoms become apparent [2,9].

Studies for this patient population are limited, with recent research mainly focusing on the safety of anesthetic management for intrathecal nusinersen administration and short-term periprocedural outcomes [10,11,12]. These studies are generally single institutions, involve small patient cohorts, and examine low-risk lumbar puncture procedures rather than longer or higher-risk surgeries. Therefore, the incidence and risk of patients with SMA undergoing more invasive procedures following the advent of new disease-modifying therapies is unknown.

Due to the paucity of large-scale procedural sedation/anesthesia outcomes in this population, we performed a retrospective cohort study using the National Surgical Quality Improvement Program-Pediatric (NSQIP-P) database to evaluate population-level perioperative trends in postoperative pulmonary complications (PPC) and length of stay (LOS) among children with SMA undergoing surgery. We hypothesized that patients with SMA have an overall elevated risk for PPC and prolonged LOS compared to the general population, but that this risk would be reduced after the introduction of disease-modifying therapies. By identifying factors associated with PPC and LOS, we aim to better define the perioperative risk profile and help guide clinical decision-making in this evolving treatment landscape.

## 2. Materials and Methods

### 2.1. Patients and Data Collection

The Ann & Robert H. Lurie Children’s Hospital of Chicago Institutional Review Board deemed this study as exempt from review, with waiver of signed patient consent (IRB 2016-89). Methods and reporting of the study adhered to the Transparent Reporting of a multivariable prediction model for Individual Prognosis or Diagnosis (TRIPOD) statement [13].

The ACS NSQIP-Pediatric registry is a prospectively collected, multicenter clinical registry which provides data on risk-adjusted outcomes to participating hospitals for the purpose of quality improvement. Sponsored by the American College of Surgeons (ACS), trained surgical clinical reviewers collect standardized, robust clinical data through in-depth chart review and phone calls to patient families [14]. Patient data includes 150 unique variables, ranging from patient demographics, preoperative risk factors, comorbidities, intraoperative factors, and outcomes until 30 days after an index procedure. Outcomes that occurred in-hospital and after discharge are recorded. Excluded surgeries include trauma, cardiac surgery, and procedures requiring cardiopulmonary bypass. Inter-rater reliability audits are conducted on all participating sites, with a disagreement rate of 5% or less being required for a site to continue participation in data submission. The combined results of these audits revealed an overall disagreement rate of approximately 2% for all assessed program variables [14]. During the study period, the ACS NSQIP-Pediatric registry included data from 50 participating hospitals in 2012, increasing to 148 hospitals by 2020, representing a mix of free-standing children’s hospitals, pediatric specialty hospitals, and pediatric units within general hospitals [14].

Patients who underwent surgery at a continuously enrolled ACS NSQIP-P hospital from 1 January 2012, to 31 December 2020 were included in this study. Cases with missing covariate or primary outcome data were excluded from the analysis. Patients with age greater than 17 years, preoperative tracheostomy, preoperative mechanical ventilation, missing covariate or primary outcome data were excluded. Patients with SMA were identified by their ICD-9 and 10 codes. The subtype of SMA was not reported and all patients with diagnosis of SMA were analyzed together. A cutoff year of 2018 was chosen for analysis of the primary outcomes as this was a full year after nusinersen received FDA approval and was in widespread use. After 2020, the variable used to identify SMA cases was no longer recorded.

### 2.2. Primary Outcome: Postoperative Pulmonary Complication

Postoperative pulmonary complication (PPC) was defined for this study as a composite outcome of postoperative pneumonia or respiratory failure requiring unplanned reintubation within seven postoperative days. This outcome was based on evidence that risk for postoperative pneumonia and postoperative reintubation rates persists up to 7 days following general anesthesia [15]. Atelectasis was not included because it is not tracked in the ACS NSQIP-P registry. The secondary outcome of interest was length of stay (LOS), defined as the number of hospital days from the day of surgery until discharge.

### 2.3. Baseline Characteristics

Baseline characteristics were collected on each patient and included the following: preoperative demographics (age, gender, race, ethnicity), surgical profile [surgical specialty, elective versus urgent or emergent surgery, operative time (z-score)], and preoperative characteristics (American Society of Anesthesiologists [ASA] physical status classification, structural pulmonary/airway abnormalities, co-existing medical history).

### 2.4. Statistical Analysis

Statistical analyses were performed on cohorts of patients undergoing surgical procedures prior to 2018 and in 2018 or after. The primary goal was to evaluate temporal trends in PPC and LOS at the population level, accounting for case complexity using available baseline and perioperative variables. Demographic and clinical characteristics were reported using means and standard deviations or median (IQR) for continuous variables and frequencies and percentages for categorical variables. Unadjusted associations for each variable and postoperative pulmonary complication were evaluated using chi-squared tests or Fisher’s exact tests for categorical variables and t-tests or Wilcoxon rank sum tests for continuous variables. To identify any association between baseline characteristics and postoperative pulmonary complication in the context of relevant perioperative variables, a multivariable logistic regression model was generated with variables being included on an a priori basis for inclusion by two independent investigators (E.C. and N.P.). Total RVU was calculated as a sum of work RVUs (Centers for Medicare and Medicaid Services Resource Based Relative Value Scale) for all current procedural terminology codes recorded for the surgery [16].

A multivariate linear regression model was used to analyze log transformed length of stay outcome with positive length of stay data included in the analysis. Collinearity diagnostics were evaluated for all variables entered into the final model using variance inflation factors. Univariable linear and logistic regression models were also used to assess for relationships between SMA and PPC, and log transformed length of stay. Results are reported as an odds ratio from logistic regression or coefficient from linear regression with 95% confidence interval (CI) with *p* values < 0.05 being considered statistically significant. All analyses were performed using R version 4.2.3 (https://www.R-project.org/; accessed on 8 August 2024).

## 3. Results

**Figure 1:**
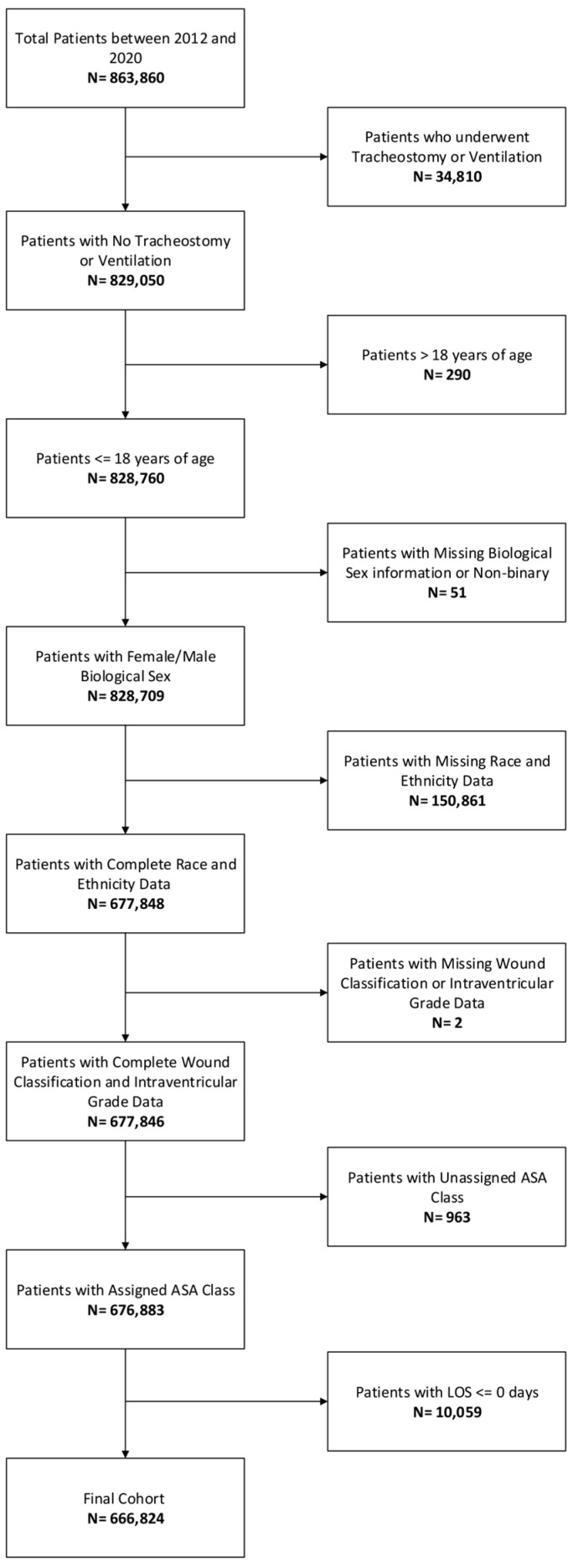
Patient inclusion and exclusion criteria. The number of cases excluded are listed below each respective criterion. ASA, American Society of Anesthesiologists.

Data for 666,824 patients were included in the final analysis. Figure 1 displays the exclusion process for the cohort as a whole. There was a total of 598 patients (0.08%) who had SMA. Among SMA cases with available anesthesia data, 90.5% underwent general anesthesia, and no cases used regional or neuraxial techniques as the primary anesthetic. The remaining 9.5% did not have a recorded anesthesia type.

### 3.1. Postoperative Pulmonary Complications (PPC): Pre-2018 vs. Post-2018

On univariable analysis, SMA was significantly associated with an increased risk of PPC prior to 2018 (OR 4.44, 95% CI 1.56–9.6, *p* = 0.008), however after 2018 it no longer had significance (OR 3.48, 0.84–9.12, *p* = 0.08, Table 1). On multivariable analysis, the association of SMA and reduction in PPC was no longer statistically significant in either time period, although the association appeared stronger prior to 2018 (OR 1.96, 95% CI 0.80–4.80, *p* = 0.14) than after 2018 (OR 1.03, 95% CI 0.33–3.26, *p* = 0.96, Table 2).

### 3.2. Length of Stay (LOS): Pre-2018 vs. Post-2018

On multivariable analysis, SMA was significantly positively associated with LOS in the pre-2018 cohort, with a coefficient from a log linear model of 0.67 (95% CI 0.32–1.01; *p* < 0.001), SMA adding an additional 1.93 days to LOS (Table 3). After 2018, the effect of SMA on LOS was no longer significant (log transformed coefficient 0.19, 95% CI −0.16–0.55; *p* = 0.28).

Prior to 2018, age was weakly inversely associated with LOS (−0.004, 95% CI −0.006 to −0.003; *p* < 0.001); post-2018, this inverse relationship became substantially stronger (−0.041, 95% CI −0.043 to −0.0039; *p* < 0.001), indicating longer hospitalizations among younger patients.

Univariable linear regression analysis showed that SMA was significantly associated with increased LOS both before and after 2018, though the effect size remained similar over time (1.769, 95% CI 1.341–2.198; *p* < 0.001; 1.711, 95% CI 1.299–2.123; *p* < 0.001; Table S1).

## 4. Discussion

Utilizing a large dataset, we found a distinct change in perioperative outcomes in patients with SMA over time. A year following widespread implementation of SMN antisense oligonucleotide therapy, the strength of association between SMA and PPC and LOS decreased, as reflected in the pre- and post-2018 cohorts. Despite not being statistically significant, SMA did have a stronger association with PPC prior to 2018 compared with after 2018 (OR of 1.96, *p* = 0.14 vs. 1.03, *p* = 0.96). Furthermore, these patients had a significantly increased length of stay, SMA leading to an additional 1.93 days. Distributions of ASA classification and surgical acuity were largely stable between pre-2018 and post-2018 cohorts (Table 1), suggesting that the observed reductions in LOS and PPC rates after 2018 are unlikely to be explained by major differences in patient comorbidity burden or case complexity.

Prior to 2018, age had a weak inverse association with LOS, with younger children expected to have slightly longer hospitalizations. After 2018, this association became substantially stronger, with younger patients experiencing longer hospitalizations in the post-nusinersen era. Although nusinersen, FDA-approved in late 2016 and widely in use by 2018, improves survival and motor outcomes when initiated early, its clinical benefits are highly dependent on treatment timing. Universal newborn screening for SMA was not widely adopted until 2018, and implementation varied by state; as a result, some infants in our 2018 or later cohort may have been diagnosed and treated after significant motor neuron loss, contributing to greater medical complexity and prolonged recovery times. Additionally, improved survival among infants with the most severe phenotypes (e.g., type 1 SMA) may have increased the proportion of medically fragile younger patients undergoing surgical procedures, further contributing to longer LOS [17]. In contrast, older children in the cohort likely represent a mix of phenotypes, including less severe, later-onset SMA (e.g., type 3) and those whose disease had stabilized after therapy initiation, which may partially explain their shorter hospitalizations relative to younger infants with more severe, early disease. As newborn screening becomes more widespread and treatment is initiated presymptomatically, future cohorts of younger patients may demonstrate improved motor outcomes, reduced perioperative resource utilization, and shorter hospitalizations.

The advent of life-altering therapies such as nusinersen and onasemnogene abeparvovec has resulted in a significant improvement in the risk profile for patients with SMA [2,9,18]. Studies regarding nusinersen (“Spinraza”) have shown improved motor milestone development and survival without permanent ventilatory support in SMA type 1, particularly when therapy has been initiated prior to the first neurological symptoms [6]. Gene replacement therapy with onasemnogene abeparvovec (“Zolgesma”) has also shown significant improvements in motor outcomes, with a large proportion of those treated not requiring permanent respiratory support [7]. Our results support the assumption that the widespread use of these FDA-approved drugs has decreased the perioperative risk profile for patients with SMA.

Despite the growing body of literature describing the safety and efficacy of anesthesia for patients with SMA undergoing routine intrathecal nusinersen administration [10,11,12], generalizability has been limited to single-institution data for only the first 24 h after a minimally invasive procedure (lumbar puncture). Data regarding extended perioperative outcomes for more invasive procedures including gastrostomy, tracheostomy tube placement, and scoliosis surgery since the introduction of disease-modifying treatments remained unknown. Limited studies prior to nusinersen and onasemnogene abeparvovec becoming widely available have reported lengthy hospitalizations and a high rate of admission to intensive care units [3,19]. Our findings of reduced PPC and LOS after introduction of antisense oligonucleotide and gene therapies support an improving risk profile at a population health level and may help clinicians better counsel families on perioperative risk and plan postoperative disposition (admission vs. home, floor vs. ICU, time of discharge).

Reduced perioperative LOS and improved risk-stratification of SMA patients for postoperative disposition in the new treatment era may also have an important impact on healthcare resource utilization and cost. A 2019 study identifying SMA patients using one of the largest administrative claims databases in the US from 2006 to 2016 found SMA patients, particularly with infantile onset, incurred significantly higher healthcare utilization and costs than the general population, with inpatient hospitalizations accounting for a significant proportion of cost [20]. As these patients who received therapy as a neonate or infant grow into children and teenagers, we will better understand their baseline respiratory and neuromuscular functions and potential reductions in healthcare utilization and cost.

Our study includes cases through December 2020, overlapping with the onset of the COVID-19 pandemic. Patient-level data on prior SARS-CoV-2 infection were not available, limiting our ability to assess its direct effect on outcomes. Pandemic-related factors, including potential respiratory sequelae, delayed surgical timing, and changes in perioperative protocols, may have influenced perioperative risk, particularly in SMA patients. Because prior infection would be expected to increase pulmonary risk and prolong LOS, the observed trend toward lower PPC rates and shorter LOS after 2018 suggests the pandemic is unlikely to explain our findings.

SMA has served as a proof of concept for antisense oligonucleotide and gene therapy, with several other disorders now in line for similar drug trials, such as amyotrophic lateral sclerosis (ALS) and Duchenne’s Muscular Dystrophy [21]. As the treatment landscape evolves, large-scale, granular datasets that capture disease subtype, treatment status, and clinical trajectory will be essential to reassessing perioperative risk, particularly for rare diseases where heterogeneity may drive markedly different outcomes. Predictive analytics has already been leveraged to forecast outcomes for rare disease groups [22]. Even with the quickly evolving prognosis for patients with SMA, such analytics may help redefine the perioperative risk profile for these patients. As health variables are not static, especially after implementation of a novel therapy, dynamic predictive models that incorporate these factors could be a useful tool for clinicians. During the COVID-19 pandemic, for example, models were developed to risk stratify patients using co-morbidities and real-time lab values [23,24]. Development of similar models for diseases in which the prognosis and risk profile has drastically changed with new therapies could provide an invaluable resource for perioperative planning and individualized patient care.

Limitations of this analysis include the relatively small number of SMA patients in this dataset (598 patients) and the lack of disease-specific details such as SMA subtype classification (types I–IV), age of disease onset, and timing of treatment initiation, which would all be valuable in more clearly delineating differences between the cohort groups. Similarly, information on treatment status (e.g., whether patients received nusinersen or gene therapy) was not available, and our interpretations regarding post-2018 trends assume that many patients were receiving treatment during this period. Differences in management strategies and improvements in respiratory care may also have contributed to some of the observed changes in perioperative outcomes. Patients in the pre-2018 cohort may have been enrolled in clinical trials assessing nusinersen or onasemnogene efficacy, which we were unable to identify. There is also limited ability to assess long-term trends, as SMA-specific outcomes are not available after 2020 due to ICD-10 code for SMA no longer being recorded by the NSQIP-P database.

Our dataset lacks information on prior SARS-CoV-2 infection, which could have influenced perioperative outcomes in the latter part of our cohort; however, any such effect would likely bias toward higher PPC rates and longer LOS, opposite to the trends we observed. Although ASA classification and surgical acuity were included in our analysis and were comparable between cohorts, other potentially relevant perioperative variables, such as regional anesthesia use, timing of extubation, and PACU admission, were not analyzed or are not captured in the NSQIP dataset. These elements, in combination with evolving perioperative practices and novel therapies, could affect both LOS and PPC incidence. Our study was designed to evaluate population-level perioperative trends rather than procedure-specific or anesthesia-specific outcomes, and future studies using targeted datasets will be important to better characterize the impact of these variables.

Finally, while our findings suggest an association between SMA and increased PPC and prolonged LOS prior to 2018, causality cannot be established due to the retrospective design of this study. However, given the profound effects of nusinersen treatment on respiratory mechanics and neuromuscular strength, particularly with early treatment initiation, it follows that PPC should be reduced following treatment.

## 5. Conclusions

Utilizing a large dataset, we found a reduced association between SMA and postoperative pulmonary complications a year following widespread implementation of SMN antisense oligonucleotide therapy, and a reduced association with LOS in the post-treatment cohort. While these findings may reflect improved motor and respiratory mechanics and the changing disease trajectory associated with the introduction of disease-modifying therapies, other factors, including advances in perioperative management and improved respiratory care strategies. Population-level data reflecting trends in the evolving clinical spectrum of patients with SMA can be used to guide further studies regarding anesthetic technique, postoperative destination, and the duration of observation following a wider spectrum of case complexity.

## Figures and Tables

**Table 1:** Univariable Logistic Regression for Postoperative Pulmonary Complications (PPC).

	Pre-2018				Post-2018			
Variables	No PPC (N = 387,719)	Yes PPC (N = 1536)	Odds Ratio (95% CI)	*p* Value	No PPC (N = 375,762)	Yes PPC (N = 1142)	Odds Ratio (95% CI)	*p* Value
Age (years)	7.63 (5.69)	5.61 (5.83)	0.94 [0.93;0.94]	<0.001	8.25 (5.83)	6.08 (5.95)	0.94 [0.92;0.95]	<0.001
SMA								
No	387,425 (99.9%)	1531 (99.7%)	Ref.	Ref.	276,424 (99.9%)	846 (99.6%)	Ref.	Ref.
Yes	294 (0.08%)	5 (0.33%)	4.44 [1.56;9.65]	0.007	296 (0.11%)	3 (0.35%)	3.48 [0.84;9.12]	0.079
Sex								
Female	168,253 (43.4%)	679 (44.2%)	Ref.	Ref.	122,699 (44.3%)	393 (46.3%)	Ref.	Ref.
Male	219,466 (56.6%)	857 (55.8%)	0.97 [0.87;1.07]	0.540	154,021 (55.7%)	456 (53.7%)	0.92 [0.81;1.06]	0.254
ASA Classification								
I	123,846 (31.9%)	68 (4.43%)	Ref.	Ref.	77,381 (28.0%)	28 (3.30%)	Ref.	Ref.
II	174,639 (45.0%)	345 (22.5%)	3.59 [2.79;4.70]	<0.001	129,270 (46.7%)	202 (23.8%)	4.30 [2.95;6.52]	<0.001
III	83,476 (21.5%)	935 (60.9%)	20.4 [16.0;26.3]	<0.001	65,905 (23.8%)	525 (61.8%)	21.9 [15.3;32.8]	<0.001
IV/V	5758 (1.49%)	188 (12.2%	59.4 [45.2;79.0]	<0.001	4164 (1.50%)	94 (11.1%)	62.1 [41.2;96.7]	<0.001
Wound Class								
Clean	186,606 (48.1%)	665 (43.3%)	Ref.	Ref.	121,595 (43.9%)	395 (46.5%)	Ref.	Ref.
Clean/Contaminated	144,282 (37.2%)	685 (44.6%)	1.33 [1.20;1.48]	<0.001	107,789 (39.0%)	362 (42.6%)	1.03 [0.90;1.19]	0.648
Contaminated	34,275 (8.84%)	88 (5.73%)	0.72 [0.57;0.90]	0.003	31,080 (11.2%)	45 (5.30%)	0.45 [0.32;0.60]	<0.001
Dirty/Infected	22,556 (5.82%)	98 (6.38%)	1.22 [0.98;1.50]	0.073	16,256 (5.87%)	47 (5.54%)	0.89 [0.65;1.20]	0.457
Septic Shock								
No	387,582 (100.0%)	1528 (99.5%)	Ref.	Ref.	276,611 (100.0%)	846 (99.6%)	Ref.	Ref.
Yes	137 (0.04%)	8 (0.52%)	15.1 [6.75;28.9]	<0.001	109 (0.04%)	3 (0.35%)	9.46 [2.25;25.1]	0.005
SIRS								
No	370,231 (95.5%)	1469 (95.6%)	Ref.	Ref.	259,662 (93.8%)	806 (94.9%)	Ref.	Ref.
Yes	17,488 (4.51%)	67 (4.36%)	0.97 [0.75;1.23]	0.793	17,058 (6.16%)	43 (5.06%)	0.82 [0.59;1.09]	0.179
Esophageal/GI Disease								
No	326,550 (84.2%)	809 (52.7%)	Ref.	Ref.	235,212 (85.0%)	491 (57.8%)	Ref.	Ref.
Yes	61,169 (15.8%)	727 (47.3%)	4.80 [4.34;5.30]	<0.001	41,508 (15.0%)	358 (42.2%)	4.13 [3.60;4.74]	<0.001
Case Type								
Elective	286,296 (73.8%)	1116 (72.7%)	Ref.	Ref.	199,721 (72.2%)	652 (76.8%)	Ref.	Ref.
Emergent	59,527 (15.4%)	250 (16.3%)	1.08 [0.94;1.23]	0.288	38,795 (14.0%)	103 (12.1%)	0.81 [0.66;1.00]	0.048
Urgent	41,896 (10.8%)	170 (11.1%)	1.04 [0.88;1.22]	0.621	38,204 (13.8%)	94 (11.1%)	0.75 [0.60;0.93]	0.048
Pulmonary Disease								
No	339,457 (87.6%)	960 (62.5%)	Ref.	Ref.	241,533 (87.3%)	511 (60.2%)	Ref.	Ref.
Yes	48,262 (12.4%)	576 (37.5%)	3.33 [3.01;3.68]	<0.001	35,187 (12.7%)	338 (39.8%)	4.54 [3.95;5.21]	<0.001
Neurological Disorder								
No	297,824 (76.8%)	766 (49.9%)	Ref.	Ref.	208,818 (75.5%)	353 (41.6%)	Ref.	Ref.
Yes	89,895 (23.2%)	770 (50.1%)	3.33 [3.01;3.68]	<0.001	67,902 (24.5%)	496 (58.4%)	4.32 [3.77;4.96]	<0.001
RVU (per unit increase)	12.4 [9.45;21.5]	25.6 [14.1;46.3]	1.02 [1.02;1.02]	<0.001	13.5 [9.45;23.1]	22.2 [12.5;38.5]	1.02 [1.01;1.02]	<0.001

PPC, postoperative pulmonary complications; SMA, spinal muscular atrophy; ASA Classification, American Society of Anesthesiologists classification; SIRS, systemic inflammatory response syndrome; GI, gastrointestinal; RVU, relative value unit; ref, reference category.

**Table 2:** Multivariable Logistic Regression for Postoperative Pulmonary Complications.

		Pre-2018		Post-2018	
Variables		Odds Ratio (95% CI)	*p* Value	Odds Ratio (95% CI)	*p* Value
Age		0.967 (0.958–0.976)	<0.001	0.968 (0.955–0.980)	<0.001
SMA		1.958 (0.799–4.800)	0.142	1.031 (0.327–3.257)	0.958
ASA Classification					
II vs. I		2.162 (1.663–2.811)	<0.001	2.805 (1.884–4.175)	<0.001
III vs. I		6.964 (5.378–9.020)	<0.001	7.703 (5.185–11.443)	<0.001
IV/V vs. I	14.428 (10.724–19.413)		<0.001	16.408 (10.513–25.608)	<0.001
Outpatient Procedure		0.149 (0.119–0.188)	<0.001	0.203 (0.157–0.263)	<0.001
Cardiac Risk Factor		1.405 (1.242–1.590)	<0.001	1.228 (1.043–1.446)	0.014
Cog Imp/Dev Delay		1.376 (1.228–1.542)	<0.001	1.808 (1.560–2.096)	<0.001
Malignancy		1.011 (0.822–1.243)	0.917	1.214 (0.931–1.583)	0.151
Structural Pulmonary Abnormality ^1^		1.746 (1.522–2.003)	<0.001	2.048 (1.730–2.424)	<0.001
RVU		1.012 (1.011–1.014)	<0.001	1.008 (1.006–1.011)	<0.001

SMA, spinal muscular atrophy; ASA Classification, American Society of Anesthesiologists classification; Cog Imp/Dev Delay, cognitive impairment/developmental delay; RVU, relative value unit. ^1^ Structural Pulmonary Abnormality = congenital or acquired anatomic abnormality of the respiratory tract, with or without respiratory compromise; includes pneumothorax or pleural effusion within 7 days of surgery and obstructive sleep apnea confirmed by sleep study.

**Table 3:** Multivariable Linear Regression for Length of Stay (LOS).

		Pre-2018		Post-2018	
Variables		Coefficient (95% CI)	*p* Value	Coefficient (95% CI)	*p* Value
Age		−0.004 (−0.006–−0.003)	<0.001	−0.041 (−0.043–−0.039)	<0.001
SMA		0.666 (0.321–1.010)	<0.001	0.194 (−0.160–0.548)	0.283
Sex					
Male vs. Female		−0.448 (−0.468–−0.429)	<0.001	−0.461 (−0.458–−0.438)	<0.001
ASA Classification					
II vs. I		1.312 (1.289–1.335)	<0.001	1.261 (1.232–1.290)	<0.001
III vs. I		2.593 (2.561–2.625)	<0.001	2.505 (2.466–2.543)	<0.001
IV/V vs. I	3.550 (3.468–3.632)		<0.001	3.570 (3.471–3.670)	<0.001
Wound Class					
Clean/Contaminated vs. Clean		0.247 (0.225–0.269)	<0.001	−0.227 (−0.254–−0.200)	<0.001
Contaminated vs. Clean		0.327 (0.287–0.367)	<0.001	−0.255 (−0.301–−0.210)	<0.001
Dirty/Infected vs. Clean		1.662 (1.617–1.707)	<0.001	1.572 (1.518–1.627)	<0.001
Septic Shock		−0.514 (−1.010–−0.018)	0.042	−0.357 (−0.935–0.222)	0.227
SIRS		0.161 (0.110–0.212)	<0.001	0.037 (−0.018–0.091	0.185
Esophageal/GI Disease		1.115 (1.086–1.143)	<0.001	0.966 (0.958–1.028)	<0.001
Case Type					
Emergent vs. Elective		1.087 (1.048–1.125)	<0.001	0.404 (0.357–0.450)	<0.001
Urgent vs. Elective		1.432 (1.393–1.470)	<0.001	0.668 (0.624–0.713)	<0.001
Pulmonary Disease		−0.129 (−0.159–−0.098)	<0.001	−0.016 (−0.053–0.021)	0.500
Neurological Disease		0.638 (0.612–0.664)	<0.001	0.363 (0.332–0.395)	<0.001
RVU		0.067 (0.067–0.068)	<0.001	0.054 (0.053–0.055)	<0.001

SMA, spinal muscular atrophy; ASA Classification, American Society of Anesthesiologists classification; SIRS, systemic inflammatory response syndrome; GI, gastrointestinal; RVU, relative value unit.

## Data Availability

Data is contained within the article.

## Supplementary Materials

The following supporting information can be downloaded at: https://www.mdpi.com/article/10.3390/children12091255/s1, Table S1: Length of Stay Univariable Linear Regression Analysis.

## Abbreviations

The following abbreviations are used in this manuscript:

ACS	American College of Surgeons
ASA	American Society of Anesthesiologists
CI	confidence interval
IRB	institutional review board
LOS	length of stay
NSQIP-P	National Surgical Quality Improvement Program Pediatric
OR	operating room
POD	postoperative day
PPC	postoperative pulmonary complication
TRIPOD	Transparent Reporting of a multivariable prediction model for Individual Prognosis or Diagnosis
