# Supplementary material for: Reduction in Perioperative Risk in Patients with Spinal Muscular Atrophy Following the Release of Disease-Modifying Therapies: An Analysis of the National Surgical Quality Improvement Program Database
Source: Children (Basel). 2025 Sep 18;12(9):1255. doi: 10.3390/children12091255 (PMC12468070; doi:10.3390/children12091255)
Supplement: Supplementary file 1 [file children-12-01255-s001.zip › children-3817973-supplementary.pdf]

Table S1: Length of Stay Univariable Linear Regression Analysis

	< 2018		≥ 2018	
**Variables**	**Odds Ratio (95% CI)**	**P Value**	**Odds Ratio (95% CI)**	**P Value**
Age	0.004 (0.002 – 0.006)	<0.001	-0.036 (-0.039 – -0.034)	<0.001
SMA	1.769 (1.341 – 2.198)	<0.001	1.711 (1.299 – 2.123)	<0.001
Sex				
Male vs Female	-0.498 (-0.522 – -0.474)	<0.001	-0.493 (-0.520 – -0.465)	<0.001
Race				
Other vs African American	-0.090 (-0.154 – -0.027)	0.006	-0.233 (-0.301 – -0.164)	<0.001
White vs African American	-0.145 (-0.179 – -0.111)	<0.001	-0.231 (-0.270 – -0.192)	<0.001
Hispanic	0.231 (0.196 – 0.266)	<0.001	0.184 (0.147 – 0.221)	<0.001
ASA Classification				
II vs I	1.621 (1.596 – 1.647)	<0.001	1.252 (1.221 – 1.283)	<0.001
III vs I	3.566 (3.535 – 3.596)	<0.001	2.953 (2.917 – 2.989)	<0.001
IV/V vs I	5.298 (5.207 – 5.390)	<0.001	4.585 (4.478 – 4.691)	<0.001
Wound Class				
Clean/Contaminated vs Clean	0.098 (0.073 – 0.124)	<0.001	-0.535 (-0.564 – -0.506)	<0.001
Contaminated vs Clean	1.136 (1.093 – 1.179)	<0.001	-0.129 (-0.174 – -0.084)	<0.001
Dirty/Infected vs Clean	2.533 (2.482 – 2.585)	<0.001	1.906 (1.848 – 1.965)	<0.001
Septic Shock	3.802 (3.187 – 4.418)	<0.001	3.024 (2.351 – 3.697)	<0.001
SIRS	1.433 (1.376 – 1.490)	<0.001	0.425 (0.369 – 0.481)	<0.001
Esophageal/GI Disease	2.314 (2.282 – 2.345)	<0.001	1.865 (1.828 – 1.903)	<0.001
Case Type				
Emergent vs Elective	2.074 (2.042 – 2.107)	<0.001	1.148 (1.109 – 1.187)	<0.001
Urgent vs Elective	2.333 (2.296 – 2.371)	<0.001	1.318 (1.279 – 1.357)	<0.001
Pulmonary	0.921 (0.885 – 0.956)	<0.001	0.831 (0.791 – 0.871)	<0.001
Neurological	1.631 (1.603 – 1.658)	<0.001	1.299 (1.268 – 1.330)	<0.001
Transfer				
Via ER vs Other	2.301 (2.275 – 2.327)	<0.001	1.580 (1.551 - 1.609)	<0.001
Hematological Disorder	2.426 (2.355 – 2.496)	<0.001	2.092 (2.024 – 2.160)	<0.001
RVU	0.068 (0.067 – 0.068)	<0.001	0.048 (0.048 – 0.049)	<0.001

SMA, spinal muscular atrophy; ASA Classification, American Society of Anesthesiologists classification; SIRS, systemic inflammatory response syndrome; GI, gastrointestinal; ER, emergency room; RVU, relative value unit.
